# Spontaneous Splenic Rupture in a Patient With Combined Congenital Factor XIII and Factor VII Deficiencies: A Case Report

**DOI:** 10.7759/cureus.105531

**Published:** 2026-03-19

**Authors:** Mohammed Abdul Muqsit Khan, Yusuf A Pathan, Mohammad Asjad, Angela A Benoj, Gayathri Pradeep, Gizalla Abdulla, Haider A Younus, Imad Nasar

**Affiliations:** 1 General Surgery, Rashid Hospital, Dubai, ARE; 2 Medicine, Sheikh Khalifa Medical City, Abu Dhabi, ARE; 3 Emergency Medicine, Ibrahim Bin Hamad Obaidullah Hospital, Ras Al Khaimah, ARE; 4 Internal Medicine, Emirates Health Services, Fujairah, ARE; 5 Medicine, Fujairah Hospital, Fujairah, ARE; 6 Orthopedics, RAK Medical and Health Sciences University, Ras Al Khaimah, ARE

**Keywords:** atraumatic splenic rupture, congenital factor vii deficiency, congenital factor xiii deficiency, factor vii, factor xiii, splenectomy

## Abstract

Spontaneous (atraumatic) splenic rupture (SSR) is an uncommon and potentially fatal emergency, most frequently linked to infection, cancer, or hematologic disorders. Although clotting factor XIII (FXIII) and factor VII (FVII) deficiencies are uncommon bleeding disorders on their own, their coexistence and association with spontaneous splenic rupture have not been reported before.

A 26-year-old adult male with known congenital FXIII deficiency, epilepsy, right-sided hemiparesis, and developmental delay presented with diffuse abdominal pain and vomiting. He was first evaluated at another hospital, where he was found to be hypotensive and tachycardic. Imaging raised concern for splenic rupture with hemoperitoneum. By the time he arrived at our center, he remained markedly tachycardic (158 bpm) but was normotensive. A FAST examination was positive for free intraperitoneal fluid, and contrast-enhanced CT demonstrated active contrast extravasation from the spleen. Laboratory evaluation demonstrated prolonged prothrombin time (20.1 s; International Normalized Ratio 1.52), normal partial thromboplastin time, FVII activity of 31% (reference values 61-199%), and later confirmed FXIII activity of 40.7% (reference range 70-140%). The patient underwent emergency laparotomy and splenectomy, with approximately two liters of hemoperitoneum identified. A planned re-look laparotomy 48 hours later showed no evidence of ongoing bleeding. His post-operative recovery was met with more challenges. He experienced pressure injuries due to prolonged immobility, ventilator-associated pneumonia, difficulty tolerating feeds, and a return of seizures. However, he gradually made progress with supportive care and time in the surgical ICU. He was extubated on postoperative day 13 and eventually recovered fully, returning home without any long-term impairments.

The underlying cause of this case, i.e., SSR in a patient with combined congenital FVII and FXIII deficiencies, makes it so remarkable. It serves as a helpful reminder that it's important to look beyond the typical suspects when someone exhibits unexplained abdominal bleeding and only minor abnormalities on standard coagulation tests. Prompt imaging, timely surgical intervention, and multidisciplinary coagulation management are essential for favorable outcomes.

## Introduction

Spontaneous (atraumatic) splenic rupture (SSR) is an uncommon but potentially fatal surgical emergency, defined as splenic hemorrhage occurring in the absence of preceding trauma [1,2]. Patients frequently present with acute abdominal pain, syncope, left shoulder tip pain, or unexplained hypotension, often mimicking common intra-abdominal pathologies and delaying diagnosis [2]. Reported etiologies of splenic rupture include infectious diseases such as Epstein-Barr virus infections and malaria, hematologic malignancies, inflammatory disorders, anticoagulant therapy, and coagulation abnormalities, with some cases remaining idiopathic despite evaluation [1].

Contrast-enhanced computed tomography (CT) is the diagnostic gold standard, allowing grading of injury using the American Association for the Surgery of Trauma (AAST) classification to guide management [1,2]. Hemodynamically stable patients with low-grade injuries, i.e., grade 1,2, and 3 in the AAST classification, may be managed conservatively with close monitoring or splenic artery embolization, whereas patients presenting with shock, active contrast extravasation, or high-grade splenic disruption generally require urgent splenectomy [1,3]. Delayed diagnosis, particularly in occult hematologic disorders, is associated with increased morbidity and mortality [1,4].

Factor XIII (FXIII) plays an essential role in the final step of coagulation. Because FXIII acts after fibrin formation, routine coagulation assays such as prothrombin time (PT) and activated partial thromboplastin time (aPTT) remain normal, making early recognition a diagnostic challenge [3]. An estimated 1 in 2 million individuals have congenital FXIII deficiency, a rare autosomal recessive condition usually caused by mutations in the FXIIIA subunit gene [3]. Clinical signs range from severe spontaneous hemorrhage, including intracranial bleeding, to delayed bleeding and poor wound healing, and the risk of bleeding is associated with FXIII levels [3]. Early recognition and FXIII replacement are essential to prevent catastrophic bleeding [3].

Factor VII (FVII) deficiency, on the other hand, is the most prevalent of the rare congenital bleeding disorders, with an estimated prevalence of one symptomatic individual per 500,000 population [5]. Isolated PT prolongation with normal aPTT is a characteristic of FVII deficiency, which is consistent with its role in the extrinsic pathway [5]. Clinical manifestations are highly variable, ranging from asymptomatic individuals to life-threatening gastrointestinal, intracranial, or postoperative bleeding. There is poor correlation between plasma FVII activity levels and bleeding severity [5]. Peri-operative bleeding is a common presentation, and optimal replacement strategies remain under investigation [5].

An increasing amount of research indicates that congenital coagulation defects may be an overlooked precipitating factor for atraumatic splenic rupture, while infections and cancers continue to be the leading causes [3,5]. Even in the absence of structural splenic disease, deficiencies affecting fibrin formation or stabilization may increase the risk of splenic parenchymal bleeding [5]. This report highlights the challenges and limitations of routine coagulation testing and the overlooked significance of comprehensive hematologic evaluation in atraumatic intra-abdominal hemorrhage.

## Case presentation

Clinical presentation

A 26-year-old adult male with a known case of congenital FXIII deficiency, developmental delay, scoliosis, chronic right-sided hemiparesis due to perinatal brain injury, and epilepsy treated with levetiracetam and carbamazepine presented with a one-day history of worsening diffuse abdominal pain accompanied by several episodes of non-bloody, non-bilious vomiting. There was no history of anticoagulant use, falls, seizures, trauma, or recent illness. After the onset of his symptoms, his parents had reported a reduction in activity and increasing discomfort.

Diagnostic evaluation

Upon initial evaluation at an outside facility, it was discovered that he had hypotension and tachycardia. A splenic capsular tear with surrounding fluid was suggested by contrast-enhanced CT, and a bedside abdominal ultrasound revealed free intraperitoneal fluid. Due to suspected intra-abdominal hemorrhage and hemodynamic instability, he was transferred to our tertiary care center.

On arrival, he appeared pale and uncomfortable but was alert. Vital signs showed a heart rate of 158 beats per minute, blood pressure of 161/141 mmHg, respiratory rate of 18 breaths per minute, temperature of 36.4°C, and oxygen saturation of 94% on room air. The Glasgow Coma Scale score was 15. Diffuse tenderness, voluntary guarding, and absent bowel sounds were noted on the abdominal examination. A focused assessment with sonography for trauma (FAST) showed free fluid in the hepatorenal and splenorenal pouches.

**Figure 1:**
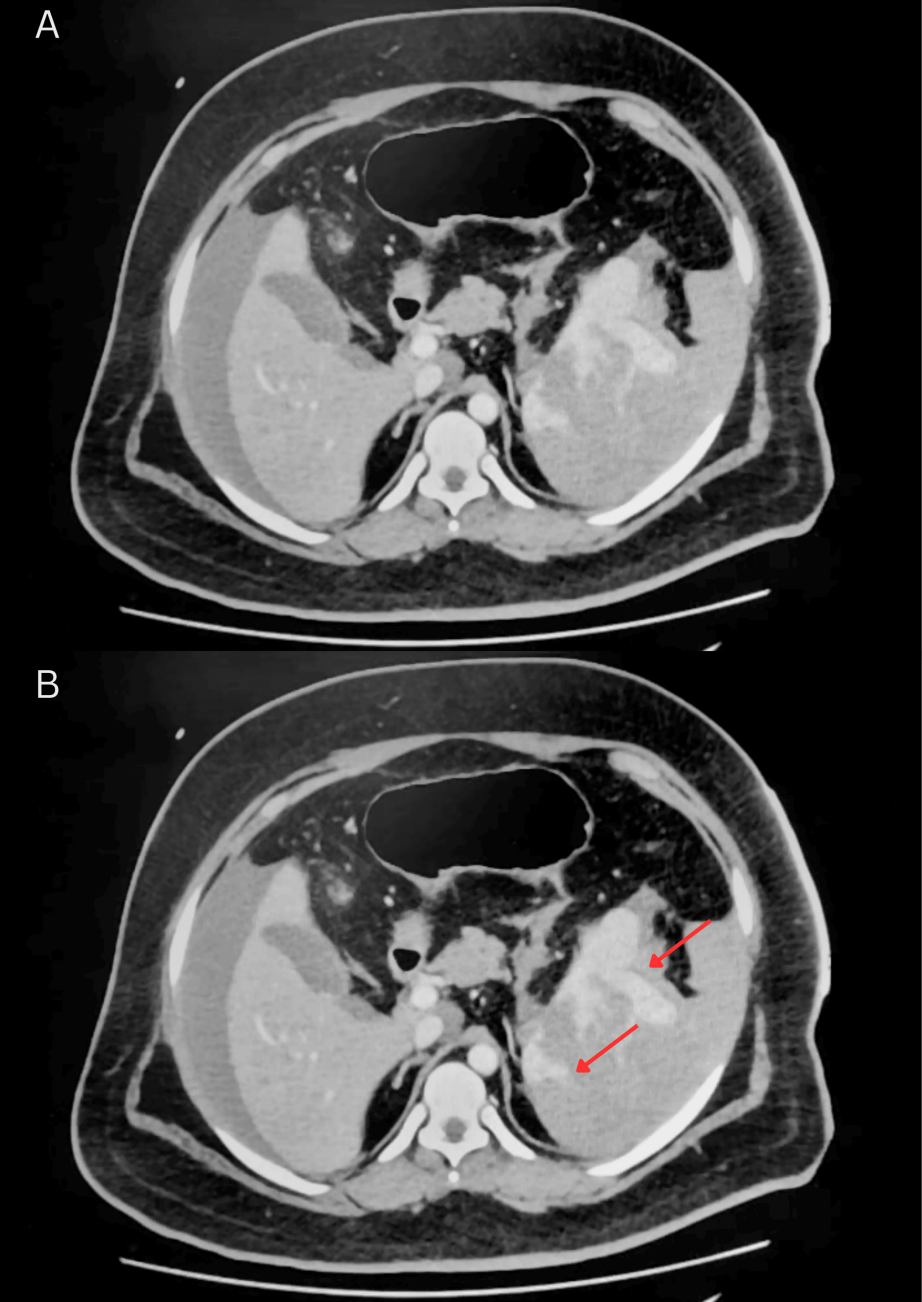
Contrast-enhanced axial venous-phase CT of abdomen demonstrating splenic rupture Pre-operative axial venous-phase contrast-enhanced CT suggestive of splenic parenchyma with focal areas of contrast extravasation (red arrows). (A) shows the unannotated scan and (B) shows the annotated scan.

**Figure 2:**
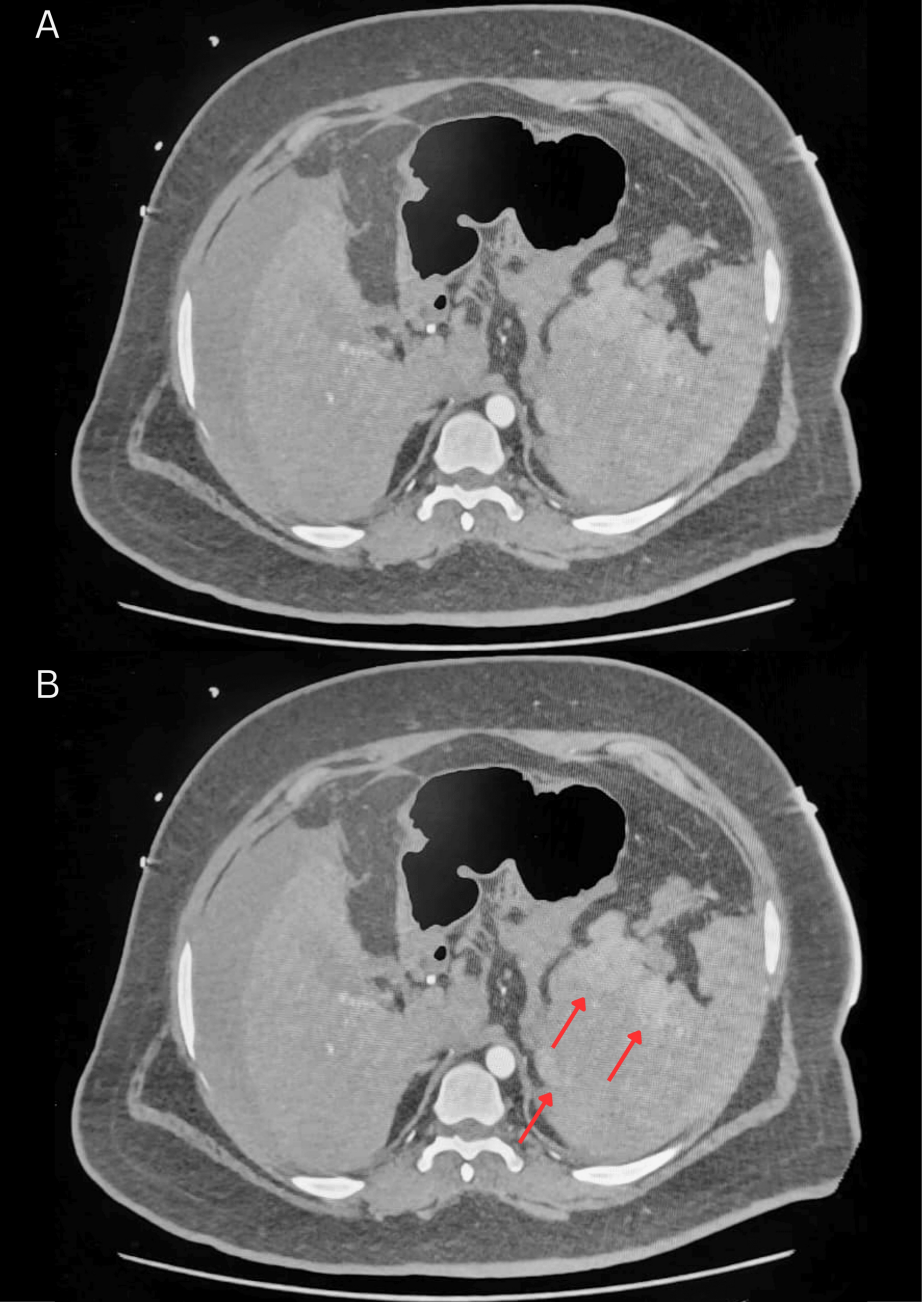
Contrast-enhanced axial arterial-phase CT of abdomen demonstrating splenic rupture Pre-operative axial arterial-phase contrast-enhanced CT suggestive of splenic parenchyma with focal areas of contrast extravasation (red arrows). (A) shows the unannotated scan and (B) shows the annotated scan.

**Figure 3:**
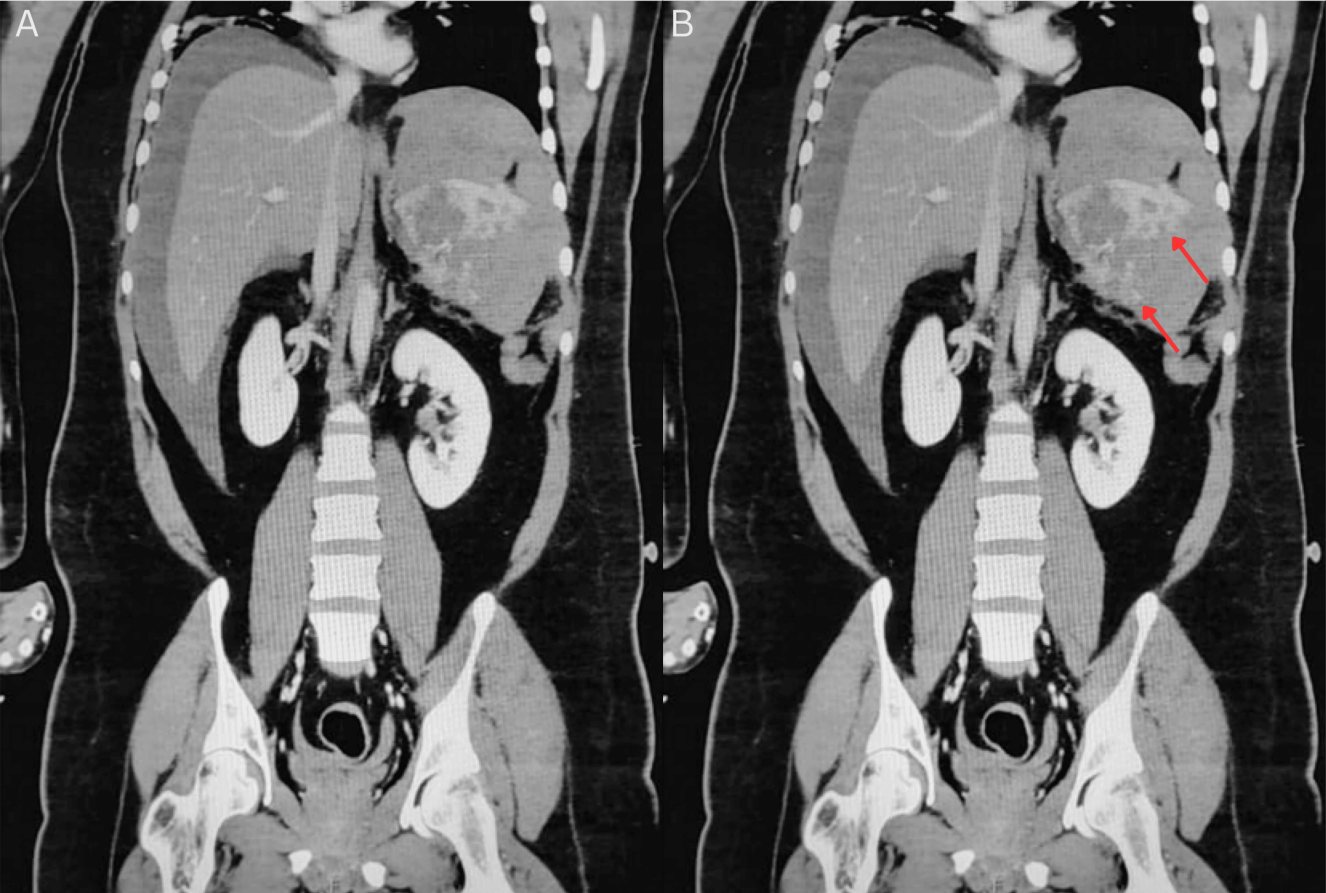
Contrast-enhanced coronal venous-phase CT of abdomen demonstrating splenic rupture Pre-operative coronal venous-phase contrast-enhanced CT suggestive of splenic parenchyma with focal areas of contrast extravasation (red arrows). (A) shows the unannotated scan and (B) shows the annotated scan.

**Figure 4:**
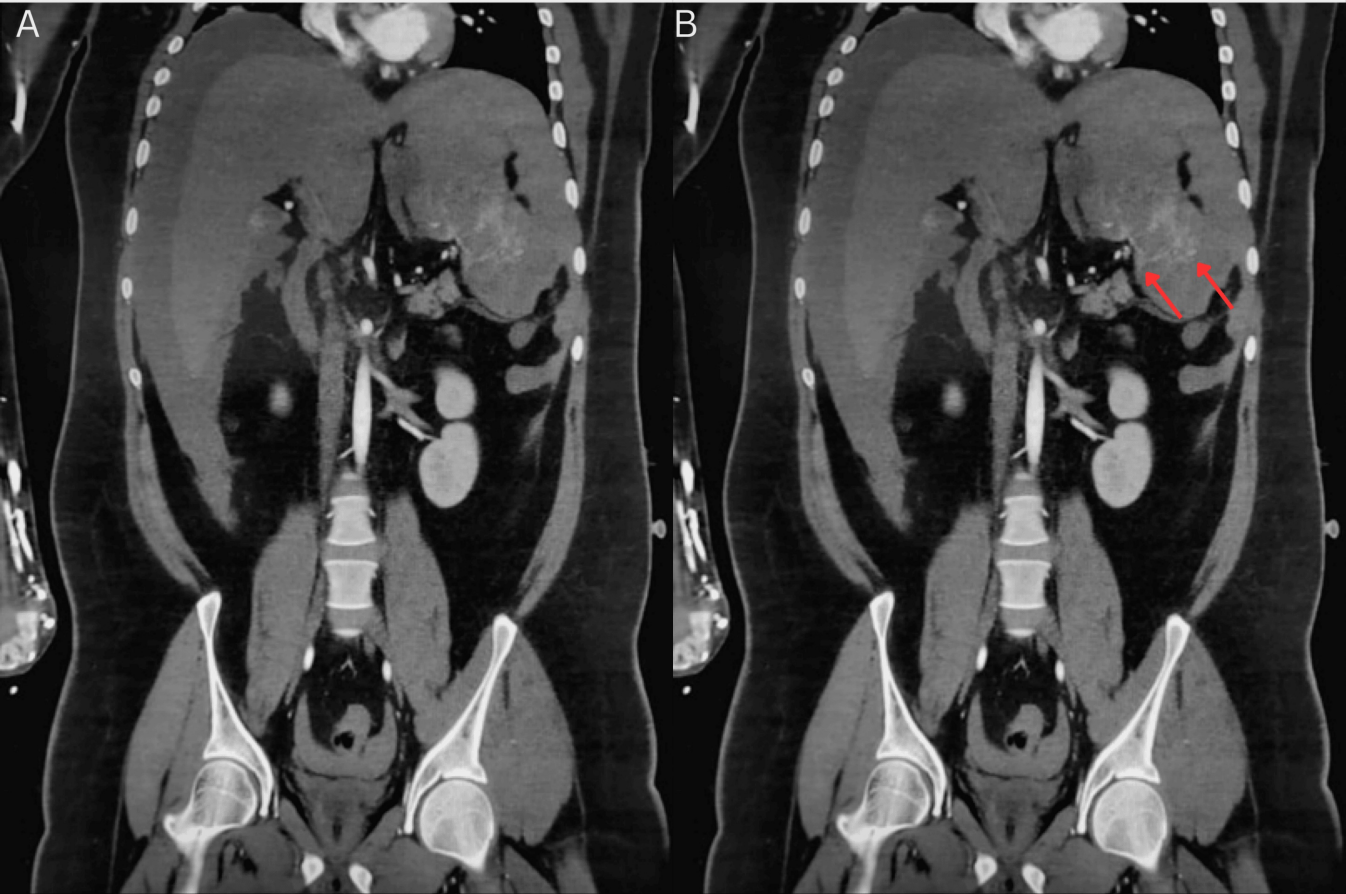
Contrast-enhanced coronal arterial-phase CT of abdomen demonstrating splenic rupture Pre-operative coronal arterial-phase contrast-enhanced CT suggestive of splenic parenchyma with focal areas of contrast extravasation (red arrows). (A) shows the unannotated scan and (B) shows the annotated scan.

CT angiography with contrast demonstrated a large volume of hemoperitoneum with active contrast extravasation along the superior splenic margin and disruption of the splenic capsule, without evidence of other solid-organ or hollow-organ injury (Figures 1-4). Laboratory studies (Table 1) revealed hemoglobin of 12.6 g/dL, hematocrit of 37.3%, platelet count of 116 × 10⁹/L, PT of 20.1 seconds (INR 1.52), and normal aPTT. Serum lactate was elevated at 2.0 mmol/L. FVII activity was 31% (reference range 61-199%), consistent with moderate deficiency. Repeat FXIII activity returned postoperatively at 40.7% (reference range 70-140%). Given the combination of active bleeding, coagulopathy, and clinical instability, emergency surgical intervention was undertaken.

**Table 1:** Laboratory investigations on arrival WBC, white blood cell; RBC, red blood cell; MCV, mean corpuscular volume; MCH, mean corpuscular hemoglobin; MCHC, mean corpuscular hemoglobin concentration; RDW, red cell distribution width; MPV, mean platelet volume; PT, prothrombin time; INR, international normalized ratio; aPTT, activated partial thromboplastin time; ABG, arterial blood gas; CRP, C-reactive protein; NT-proBNP, N-terminal pro–B-type natriuretic peptide.

Investigation	Result	Reference Range
FBC (CBC + AUTO DIFF)		
WBC count	17.8	3.6 - 11 × 10³ cells/µL
RBC count	4.42	4.50 - 5.50 × 10⁶ cells/µL
Hemoglobin	12.6	13.0 - 17.0 g/dL
Hematocrit	37.3	40.0 - 50.0 %
MCV	84.4	77.0 - 95.0 fL
MCH	28.5	27.0 - 32.0 pg
MCHC	33.8	31.5 - 34.5 g/dL
RDW	14.6	11.5 - 14.0 %
Platelets	116	150 - 410 × 10³ cells/µL
MPV	8.8	7.4 - 10.4 fL
Neutrophils absolute	14.80	2.00 - 7.00 × 10³ cells/µL
Lymphocytes absolute	1.80	1.00 - 3.00 × 10³ cells/µL
Monocytes absolute	1.30	0.20 - 1.00 × 10³ cells/µL
Eosinophils absolute	0.00	0.00 - 0.50 × 10³ cells/µL
Basophils absolute	0.00	0.00 - 0.10 × 10³ cells/µL
Neutrophil %	82.9	
Lymphocyte %	9.9	
Monocyte %	7.1	
Eosinophil %	0.0	
Basophil %	0.1	
MDW	22.00	<20.00
Nucleated RBCs	0	0 per 100 WBC
Coagulation Panel		
Prothrombin time	20.1	12.6 - 16.3 seconds
INR	1.52	0.8 - 1.1
PT Control	13.7	
aPTT	32.6	28 - 41 seconds
aPTT Control	36.8	
Factor 7 Assay (VII)	31	61 - 199 %
Fibrinogen	348	200 - 400 mg/dL
ABG Metabolic Panel		
pH	7.337	7.35 - 7.45
PCO2	36.2	35 - 45 mmHg
PO2	266	83 - 108 mmHg
HCO3	19.4	21 - 28 mmol/L
CTHB	11.7	13.0 - 18.0 g/dL
SO2	100.3	95 - 99 %
FO2HB	98.0	95 - 98 %
FCOHB	1.4	0.5 - 1.5 %
K	3.7	3.4 - 5.4 mmol/L
Na	143	134 - 143 mmol/L
Cl	108	98 - 108 mmol/L
Phosphate	3.1	2.5 - 4.5 mg/dL
Ionized calcium	1.15	1.15 - 1.29 mmol/L
Glucose	133	60 - 100 mg/dL
Lactate	2.0	0.5 - 1.6 mmol/L
Base (ECF) (-)	6.5	
Anion Gap	16	6 - 14 mmol/L
Plasma Lactic Acid	2.81	0.5 - 2.2 mmol/L
Renal Profile		
Urea	29	12 - 40 mg/dL
Creatinine	0.93	0.70 - 1.20 mg/dL
eGFR	116.1	> 60 mL/min/1.73 m²
Liver Profile		
Bilirubin total	1.06	0 - 1.2 mg/dL
SGPT (ALT)	20	0 - 41 U/L
SGOT (AST)	22	0 - 40 U/L
ALP	71	40 - 129 U/L
Total protein	6.6	6.6 - 8.7 g/dL
Albumin	4.0	4.4 - 5.1 g/dL
Globulin	2.6	2.8 - 3.4 g/dL
Pancreatic profile		
Lipase	11	13 - 60 U/L
Inflammatory markers		
CRP	94.9	<5.0 mg/dL
Cardiac biomarkers		
Troponin T	78	<14 ng/L
NT - proBNP	343	<125 pg/L

Operative findings and procedure

Midline laparotomy revealed approximately two liters of hemoperitoneum. The spleen was severely fragmented with multiple capsular disruptions and active bleeding, consistent with spontaneous atraumatic rupture. No additional intra-abdominal injuries were identified. Splenectomy was performed with hilar control using a vascular stapler. Hemostasis was achieved with electrocautery and packing, and a closed-suction drain was placed before closure. Intra-operatively, two units of packed red blood cells, two units of fresh frozen plasma, and one unit of cryoprecipitate were transfused.

Post-operative course

The patient was transferred to the surgical intensive care unit (ICU) while intubated. A planned re-look laparotomy performed 48 hours later showed no active bleeding. His ICU course was complicated by ventilator-associated pneumonia due to *Pseudomonas aeruginosa*, pressure injuries, feeding intolerance necessitating temporary parenteral nutrition, and recurrent seizures requiring adjustment of his anti-epileptic management. Over time, his clinical condition improved, allowing for extubation by day 13 post-op. He gradually returned to baseline in terms of mobility and oral intake and was discharged home in stable condition with hematology follow-up and post-splenectomy vaccination planning.

## Discussion

Overview of SSR

SSR is a rare but potentially life-threatening surgical emergency, most commonly associated with infections, hematologic malignancies, inflammatory disorders, and coagulation abnormalities [6,7]. Unlike traumatic splenic injury, patients often present without a history of trauma and with nonspecific symptoms such as abdominal pain, syncope, or shoulder tip pain, which can delay diagnosis [8,9].

Comparison with previously reported cases

In the literature (Table 2), Case 1 describes an adult patient presenting with sudden abdominal pain and hypotension requiring emergency splenectomy despite having no underlying coagulopathy [10]. Similar to our patient, both presented with sudden abdominal pain and hemodynamic instability necessitating surgical intervention; however, unlike Case 1, our patient had a documented underlying coagulopathy, which likely contributed to the hemorrhagic event.

**Table 2:** Clinical characteristics and management of reported cases of spontaneous splenic rupture FXIII, Factor XIII; FVII, Factor VII.

Case	Patient Age/Demographics	Presentation	Underlying Cause/Coagulopathy	Management	Key Points/Learning
Case 1 [10]	Adult	Sudden abdominal pain, hypotension	None identified	Emergency splenectomy	Spontaneous rupture can occur without predisposing conditions
Case 2 [8]	Young patient	Acute abdominal pain, hemodynamic instability	Not specified	Supportive care, close monitoring	Early imaging and individualized management are crucial
Case 3 [6]	Not specified	Spontaneous abdominal pain, hypotension	FXIII deficiency (coagulopathy)	Surgery after recognition of FXIII deficiency	Routine coagulation tests may not detect FXIII deficiency; coagulopathy often diagnosed post-event
Case 4 [7]	Adult	Atraumatic abdominal pain	Not specified	Depends on hemodynamic status (conservative or surgical)	Highlights spontaneous rupture in otherwise healthy individuals
Case 5 [9]	16-year-old male	Grade III splenic hemorrhage, hemodynamically stable	FVII deficiency, Noonan syndrome	Conservative management (transfusions, factor replacement)	Congenital coagulation disorders can precipitate rupture even if initially stable

Case 2 reported a young patient with acute abdominal pain and hemodynamic instability successfully managed with supportive care and close monitoring [8]. Our patient presented acutely and required rapid imaging, but differed in that conservative management was not feasible due to persistent hemodynamic instability, whereas case 2 remained stable under observation without requiring active surgical intervention.

Case 3 illustrated spontaneous rupture in a patient with FXIII deficiency, where normal routine coagulation tests delayed recognition of the underlying bleeding disorder [6]. Our patient was similar in that the rupture occurred in the setting of a coagulation factor deficiency, and that standard tests alone would have been insufficient for diagnosis; however, unlike Case 3, part of our patient’s coagulopathy (FXIII deficiency) was already known prior to the event, allowing earlier consideration of bleeding risk.

In Case 5, a 16-year-old male with Noonan syndrome and FVII deficiency presented with a grade III splenic hemorrhage and was managed conservatively [9]. In contrast, our patient, despite also having coagulation deficiencies, required emergency splenectomy due to hemodynamic instability, representing a more severe clinical course compared to the conservatively managed Case 5.

In our patient, the presentation of sudden abdominal pain, hypotension, and radiologic evidence of splenic rupture was similar to the previously reported cases, likely due to underlying coagulation defects [6,9], while also demonstrating a unique combination of multiple factor deficiencies and the need for immediate surgical intervention, distinguishing our report from prior cases.

Differential diagnosis of SSR

Several differential diagnoses were considered at presentation, including perforated hollow viscus, acute pancreatitis, hepatobiliary sepsis, ruptured visceral artery aneurysm, infectious splenomegaly, and hematologic malignancy. These were systematically excluded as CT imaging showed no gastrointestinal perforation or pneumoperitoneum; pancreatic enzymes were within normal limits; imaging revealed no biliary pathology; there was no evidence of aneurysmal bleeding; infectious work-up was negative; and peripheral blood evaluation showed no hematologic malignancy. The rapid decline in hemoglobin alongside a discrete splenic hematoma on contrast-enhanced CT confirmed the diagnosis of splenic rupture as the cause of shock [8,10].

Role of coagulation abnormalities in SSR

**Figure 5:**
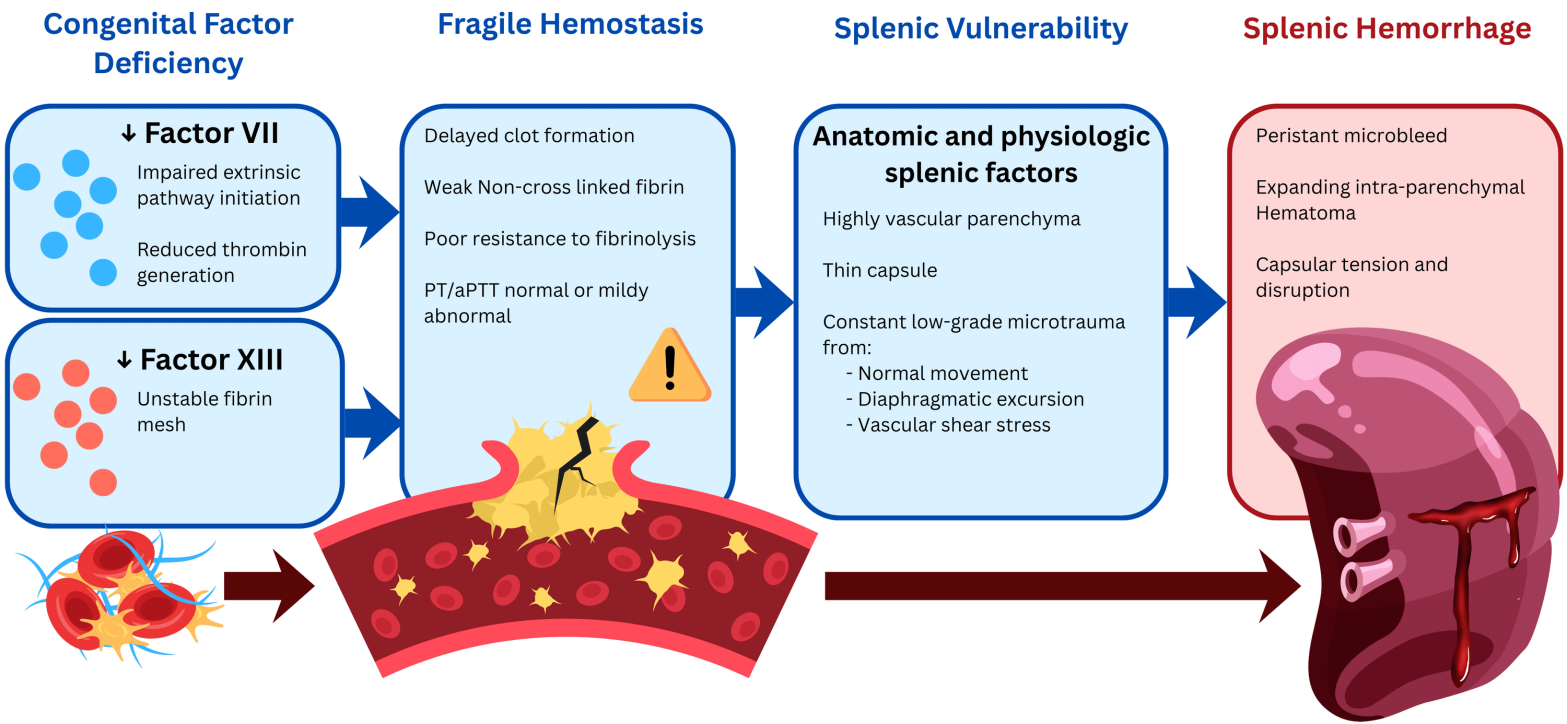
Pathophysiology of spontaneous splenic rupture in combined factor XIII and factor VII deficiencies PT, prothrombin time; aPTT, activated partial thromboplastin time. The image is an original illustration conceived and created by the author using Procreate and Canva; no external template was used. Illustrated by: Yusuf Arshad Pathan.

Coagulopathy is a recognized risk factor for atraumatic splenic hemorrhage, particularly deficiencies of clotting factors such as FXIII and FVII [6,9]. FXIII is essential for fibrin cross-linking and clot stability, and its deficiency can result in delayed or spontaneous internal bleeding despite normal routine coagulation parameters [6]. In contrast, FVII deficiency usually presents with a prolonged PT and may be associated with syndromic conditions such as Noonan syndrome (Figure 5) [9]. Unlike Cases 3 and 5, where coagulopathy was diagnosed only after the hemorrhagic event, our patient experienced splenic rupture as the initial manifestation of a previously diagnosed FXIII deficiency and a newly diagnosed FVII deficiency.

Diagnosis and classification of SSR

**Figure 6:**
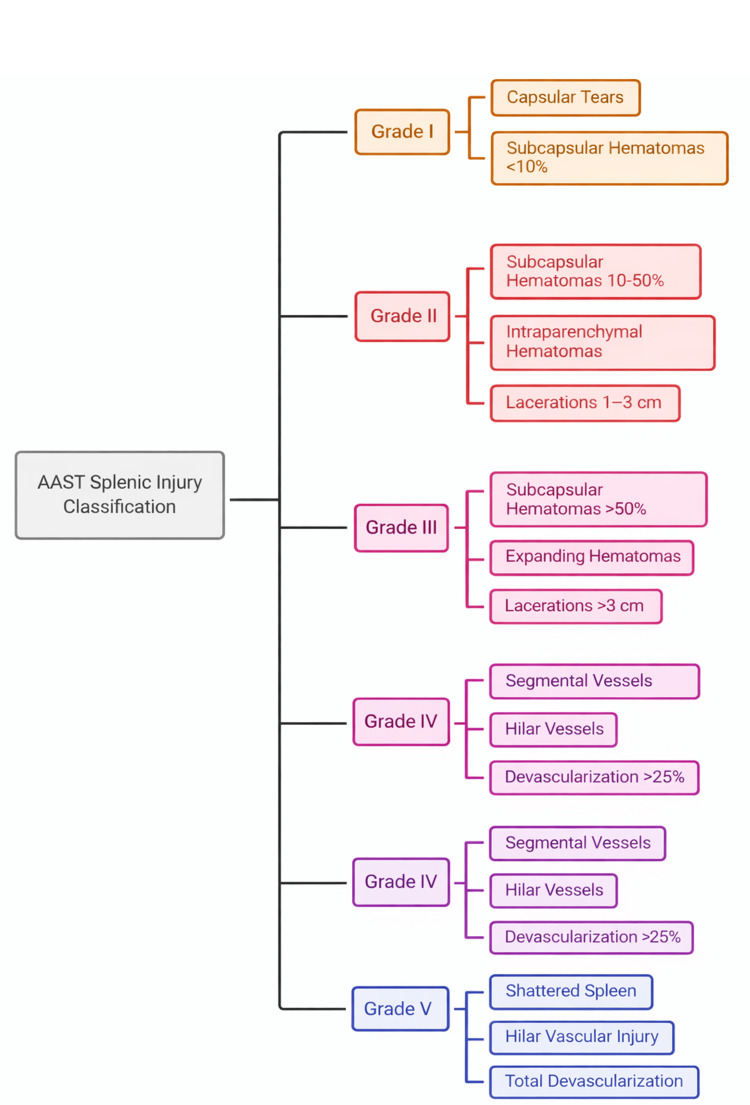
American Association of the Surgery of Trauma (AAST) splenic injury classification The AAST grading system stratifies splenic injury from grade I to grade V according to the extent of capsular disruption, hematoma burden, parenchymal laceration and vascular compromise. Illustration was created by the author using Microsoft Word with information from the book by Bailey and Love [4]. Illustrated by: Mohammed Abdul Muqsit Khan.

Contrast-enhanced CT is the gold standard for diagnosing and grading splenic injury [9,10], and the American Association for the Surgery of Trauma (AAST) grading system (Figure 6) is widely used to guide management, even in non-traumatic cases [8,9]. Active contrast extravasation on CT predicts failure of conservative therapy and the need for intervention [8,10]. In our patient, CT demonstrated a high-grade injury with hemodynamic instability, favoring operative management, similar to interventions described in Cases 1 and 3. The absence of alternative intra-abdominal pathology on imaging further supported the decision to proceed with definitive splenic intervention rather than conservative observation.

Management of spontaneous splenic rupture must be individualized based on the underlying etiology, radiologic grade, and hemodynamic status [7,8]. Hemodynamically stable patients with grade I-III injuries have been successfully managed with observation and transfusion alone [8,9]. However, splenectomy or angio-emboliszation is typically required in patients presenting with shock, ongoing transfusion requirement, or active bleeding [6,10]. In our patient, persistent instability necessitated emergency splenectomy, consistent with management strategies described in severe presentations in Cases 1 and 3.

Peri-operative management of SSR

Peri-operative correction of the coagulation defect is a critical component of management [6,9]. Replacement with specific factor concentrates, fresh frozen plasma, vitamin K, and tranexamic acid has demonstrated variable efficacy depending on the underlying deficiency [6,9]. Delayed recognition of coagulopathy can increase peri-operative morbidity, as highlighted in Case 3, where FXIII deficiency was diagnosed only after repeated bleeding episodes. In our patient, early hematology involvement enabled targeted replacement therapy, contributing to favorable postoperative recovery.

The association between congenital or acquired factor deficiencies and SSR remains under-recognized [6,9]. Most large reviews of atraumatic rupture cite infection and malignancy as predominant causes, with factor deficiencies rarely reported [7]. This underscores the importance of comprehensive coagulation evaluation in all non-traumatic splenic hemorrhages, particularly for FXIII deficiency, which may not be detected by standard PT or aPTT testing [6].

Although guidelines such as those from the AAST and the World Society of Emergency Surgery (WSES) are primarily derived from traumatic splenic injury and may not fully address spontaneous cases [9], their application to atraumatic rupture has been successful in several reports [8,9]. Our experience supports the use of trauma-based grading systems while emphasizing that definitive management should also be guided by the underlying pathology, particularly coagulopathy [6,9].

Post-splenectomy care, including vaccination and long-term hematology follow-up, is essential to prevent overwhelming post-splenectomy infection and recurrent bleeding [6,10]. Patients with inherited factor deficiencies require lifelong counseling and prophylaxis before invasive procedures [6,9]. Our patient was discharged with appropriate immunization and targeted factor replacement, in line with previously described protocols.

This case illustrates that SSR may be the first manifestation of an occult coagulation disorder [6,9]. Early CT imaging, a high index of suspicion, and multidisciplinary care are critical for survival [8,10]. Routine screening for coagulation factor deficiencies, particularly FXIII and FVII, should be considered in all atraumatic splenic hemorrhages [6,9].

## Conclusions

SSR is an uncommon but catastrophic cause of an acute abdomen, and diagnosis may be delayed in the absence of trauma or obvious predisposing factors. This case highlights atraumatic splenic rupture in a young adult with congenital FXIII and FVII deficiencies, demonstrating the abrupt life-threatening presentation of inherited bleeding disorders. Profound bleeding may occur despite mild abnormalities in standard tests.

A successful outcome depends on early imaging, prompt surgical intervention, and close coordination with hematology. This case expands the range of hematological disorders associated with spontaneous splenic rupture. Multidisciplinary management remains essential to improving outcomes in these rare but life-threatening presentations.
